# Current achievements of nanoparticle applications in developing optical sensing and imaging techniques

**DOI:** 10.1186/s40580-016-0090-x

**Published:** 2016-11-14

**Authors:** Jong-ryul Choi, Dong-Myeong Shin, Hyerin Song, Donghoon Lee, Kyujung Kim

**Affiliations:** 1 Medical Device Development Center, Daegu-Gyeongbuk Medical Innovation Foundation (DGMIF), Daegu, 41061 Republic of Korea; 2 Research Center for Energy Convergence Technology, Pusan National University, Busan, 46241 Republic of Korea; 3 Department of Cogno-Mechatronics Engineering, Pusan National University, Busan, 46241 Republic of Korea; 4 Department of Psychology, Pusan National University, Busan, 46241 Republic of Korea

**Keywords:** Surface plasmons, Field localization, Nanoparticles, Optical sensing, Optical imaging

## Abstract

Metallic nanostructures have recently been demonstrated to improve the performance of optical sensing and imaging techniques due to their remarkable localization capability of electromagnetic fields. Particularly, the zero-dimensional nanostructure, commonly called a nanoparticle, is a promising component for optical measurement systems due to its attractive features, e.g., ease of fabrication, capability of surface modification and relatively high biocompatibility. This review summarizes the work to date on metallic nanoparticles for optical sensing and imaging applications, starting with the theoretical backgrounds of plasmonic effects in nanoparticles and moving through the applications in Raman spectroscopy and fluorescence biosensors. Various efforts for enhancing the sensitivity, selectivity and biocompatibility are summarized, and the future outlooks for this field are discussed. Convergent studies in optical sensing and imaging have been emerging field for the development of medical applications, including clinical diagnosis and therapeutic applications.

## Introduction

Since the first conceptual discussion by Richard Feynman in 1959 and the first use by Norio Taniguchi in 1974 [1], nanotechnology has been consistently developed and applied in various scientific and industrial areas [2–4]. Nanostructures from zero to three dimensions [5–14] have recently received considerable attention due to their remarkable physical properties and potential applications. For instance, semiconductor nanocrystals named quantum dots (QDs) have been employed to develop larger and flexible display components [15–18]. Carbon nanotubes (CNTs) have extraordinary mechanical and electrical properties [19, 20] and have been used as high-performance components in electronics and material science [21–23]. Recent applications of nanotechnology and nanomaterials have extended to biomedical fields, for example, efficient drug delivery [24–26], non-viral gene therapy [27, 28] and nanomaterial-assisted tissue culture engineering [29, 30].

Metallic nanostructures using gold, silver, and aluminium have been widely applied to improve sensing and imaging techniques relevant to plasmonics [31–33]. There have been many reports that plasmonic applications show a bright future in highly sensitive and highly resolved optical measurement systems [34–36]. Among the metallic nanostructures, nanoparticles have been widely applied in numerous optical measurement systems because many synthetic methods have been developed and the surface of the nanoparticles can be easily modified using chemical approaches [37, 38]. Additionally, the size is easily controllable, and the nanoparticles can be synthesized to be degradable somehow in vivo [39, 40]. Although plasmonics-based applications of nanoparticles have many advantages to enhance the optical measurement systems, they still need more investigations of their stability, compatibility, and uniformity. Moreover, novel approaches are needed for additional enhancements in the sensitivity and resolution of the optical sensing and imaging methods, respectively.

In this review, we introduce varied approaches to improve the performance of optical measurement techniques using metallic nanoparticles in sensing and imaging. We first discuss the plasmonic effects on the surface of the nanoparticles through a theoretical approach based on the Maxwell equation, and then we consider various applications for sensitivity enhancements in biosensing and imaging methods that have been investigated in the last decades. Moreover, we address recent research interests: (1) specially designed nanoparticles for improved performance, (2) multi-material nanoparticle synthesis, (3) biocompatibility-enhanced nanoparticles, and (4) combinations of nanostructures and nanoparticles based on co-plasmonic enhancements. Consequently, we suggest future research directions based on multi-functional nanoparticles in the development of high-performance optical measurement systems.

## Plasmonic nanoparticles for optical sensing and imaging applications

### Localized surface plasmon resonance biosensors based on nanoparticles

Since the original theoretical investigations and experimental confirmations of electromagnetic fields travelling on a metal-dielectric interface, which were named surface plasmon polaritons (SPPs) [41], the maximum excitation of the SPPs under specific conditions, which was defined as surface plasmon resonance (SPR), and its applications such as photonic circuits and nano-photonic devices have been established [42–45]. According to Maxwell’s equations, the dispersion relation of surface plasmon waves can be derived with the boundary conditions determined from the interface of the metal and dielectric layers as described below:

$$ k_{SP} = \frac{\omega }{c}\sqrt {\frac{{\varepsilon_{\text{metal}} \varepsilon_{\text{dielectric}} }}{{\varepsilon_{\text{metal}} + \varepsilon_{\text{dielectric}} }}} $$

where *ε*_metal_ and *ε*_dielectric_ are the permittivity of the metal and dielectric layers, respectively, *ω* is the angular frequency of the incident light, and *c* is the speed of light. *k*_*SP*_ is the surface momentum parallel to the interface between the metal and dielectric layers [46].

In the case of SPR on nanoparticles in the dielectric surrounding, the electron densities on nanoparticles can be coupled to electromagnetic fields radiated with wavelengths that are larger than the sizes of the nanoparticles. The scattering and absorbance probabilities for metallic perfectly spherical nanoparticles can be determined as:

$$ \sigma_{\text{scattering}} = \frac{8\pi }{3}k^{4} r^{6} \left| {\frac{{\varepsilon_{\text{metal}} - \varepsilon_{\text{dielectric}} }}{{\varepsilon_{\text{metal}} + 2\varepsilon_{\text{dielectric}} }}} \right|^{2} $$

$$ \sigma_{\text{absorbance}} = 4\pi kr^{3} {\text{Im}}\left[ {\frac{{\varepsilon_{\text{metal}} - \varepsilon_{\text{dielectric}} }}{{\varepsilon_{\text{metal}} + 2\varepsilon_{\text{dielectric}} }}} \right] $$

where *k* is the wavenumber of the incident light, *ε*_metal_ and *ε*_dielectric_ indicate the permittivity of the metallic nanoparticles and the dielectric surrounding, respectively, and *r* is the radius of the nanoparticles [47, 48]. Resonance occurs when *ε*_metal_ = −2*ε*_dielectric_, in which the incident light is totally scattered by the nanoparticles.

**Fig. 1:**
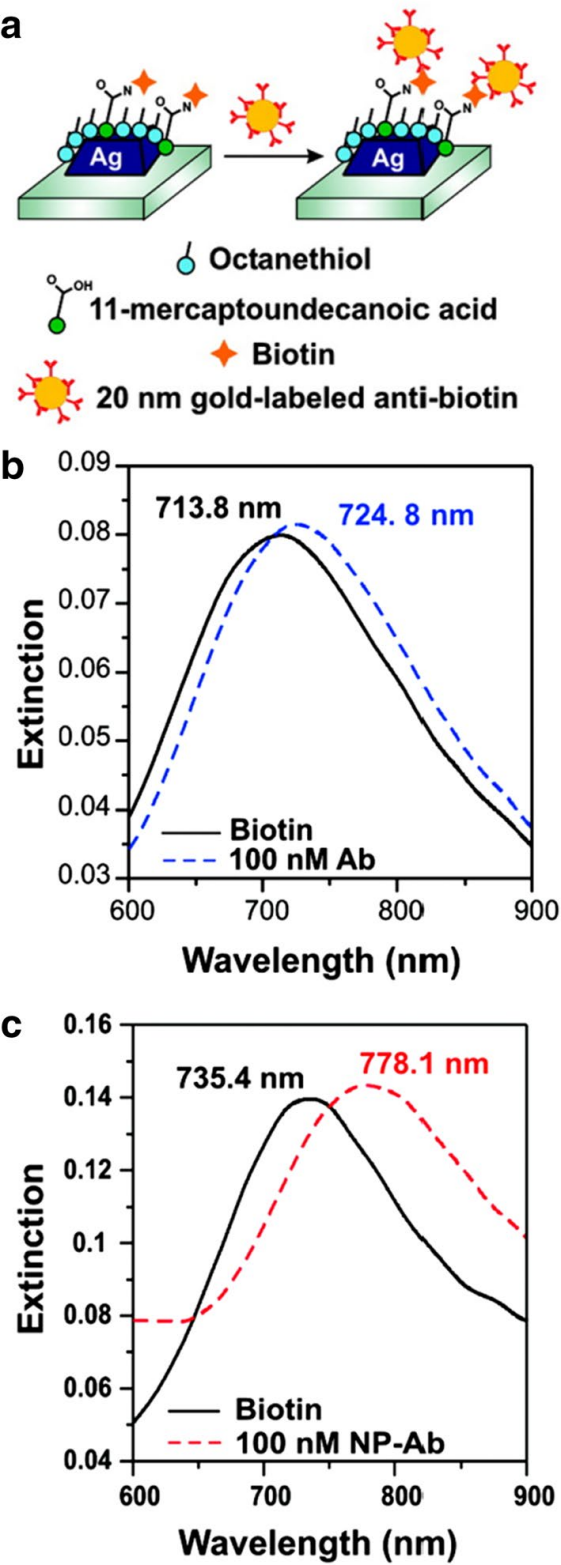
LSPR biosensors with wavelength shift amplifications using gold nanoparticle-attached antibodies. **a** Experimental schematic of biosensing. **b** LSPR spectral extinction with binding non-labelled and **c** gold nanoparticle labelled antibiotins. The enhancement of the sensing efficiency (a spectral shift) is by a factor of 3.88 by using labelled gold nanoparticles. The re-print of this figure from [55] is permitted by the American Chemical Society

A localized surface plasmon resonance (LSPR), one of the several types in the SPR phenomenon, is defined as extremely accumulated and enhanced surface plasmons between metallic nanoscale components, including nanoparticles, nanoscale posts, and dielectrics [49–52]. Because the condition at LSPR is sensitively changed depending on the surface properties of the nanoparticles, LSPR has been applied to develop highly sensitive chemical, biological and medical sensors. Before the introduction of several studies of LSPR based molecular sensing applications, theoretical comparison of SPR on metallic film propagations and LSPR on metallic nanoparticles is described. From the equations in previous paragraphs, a sensing response (*R*_sensing_) on a metallic film (general surface plasmon resonance) or nanoparticle (LSPR) with a molecular attachment can be derived as

$$ R_{\text{sensing}} = A{\Delta }n\left(1 - e^{{\frac{ - 2d}{{l_{d} }}}} \right) $$

which *A* is refractive index sensitivity, Δ*n* is a difference of refractive index between a molecule-attached layer and an environmental media, *d* is a thickness of the attached layer, and *l*_*d*_ is an electromagnetic plasmonic decay length. In the previous study by Svedendahl et al. [53], surface plasmonic resonance molecular sensing by metallic film propagations has higher *A*, but LSPR by metallic nanoparticles has extremely lower *l*_*d*_. Therefore, sensitivities of surface plasmon and LSPR are similar. On the other hand, extremely lower *l*_*d*_ in LSPR provides higher selectivity and signal-to-noise ratio (SNR) to detect molecular binding because the effect of refractive index variances in the superstrate via temperature or buffer condition changes is proportional to *l*_*d*_. For this reason, several research groups applied LSPR using metallic nanoparticles to biomolecular binding including biotin–streptavidin attachments. Metallic nanoparticles with binding probes that capture the specific analytes are generally employed in LSPR sensors. Fujiwara et al. [54] developed surface-modified gold nanoparticles to detect and monitor serum albumins using LSPR spectroscopic biosensors. Gold nanoparticles with biochemical probes for binding bovine/human serum albumin were functionalized on a glass substrate, and the LSPR of the gold nanoparticles enables the enhancement of the spectroscopic sensing of the analyte binding process. Paige Hall et al. [55] demonstrated gold-nanoparticle-based LSPR biosensors to dramatically amplify the shift of the resonance wavelength. By the detection of analytes adhered on a surface of gold nanoparticles labelled with antibodies, 400% enhancements in the LSPR spectroscopic sensing of biological analytes was achieved, as shown in Fig. 1. Li et al. [56] introduced the use of polyelectrolyte-functionalized gold nanoparticles in LSPR bio-sensing. In the study of biotin–streptavidin binding, the streptavidin detection limit of the binding was 100 ng, with a red-shift of 1.5 nm, and the feasibility to employ highly sensitive LSPR biosensors was suggested. Obare et al. [57] investigated lithium ion sensors using gold nanoparticles with ligands that can bind to Li^+^. The detection of lithium ions was successfully achieved in a high sensitivity manner by measuring the spectrum of the gold nanoparticle aggregations that result from the ligand-Li^+^ binding.

### Nanoparticle-enhanced Raman spectroscopic sensing and imaging techniques

**Fig. 2:**
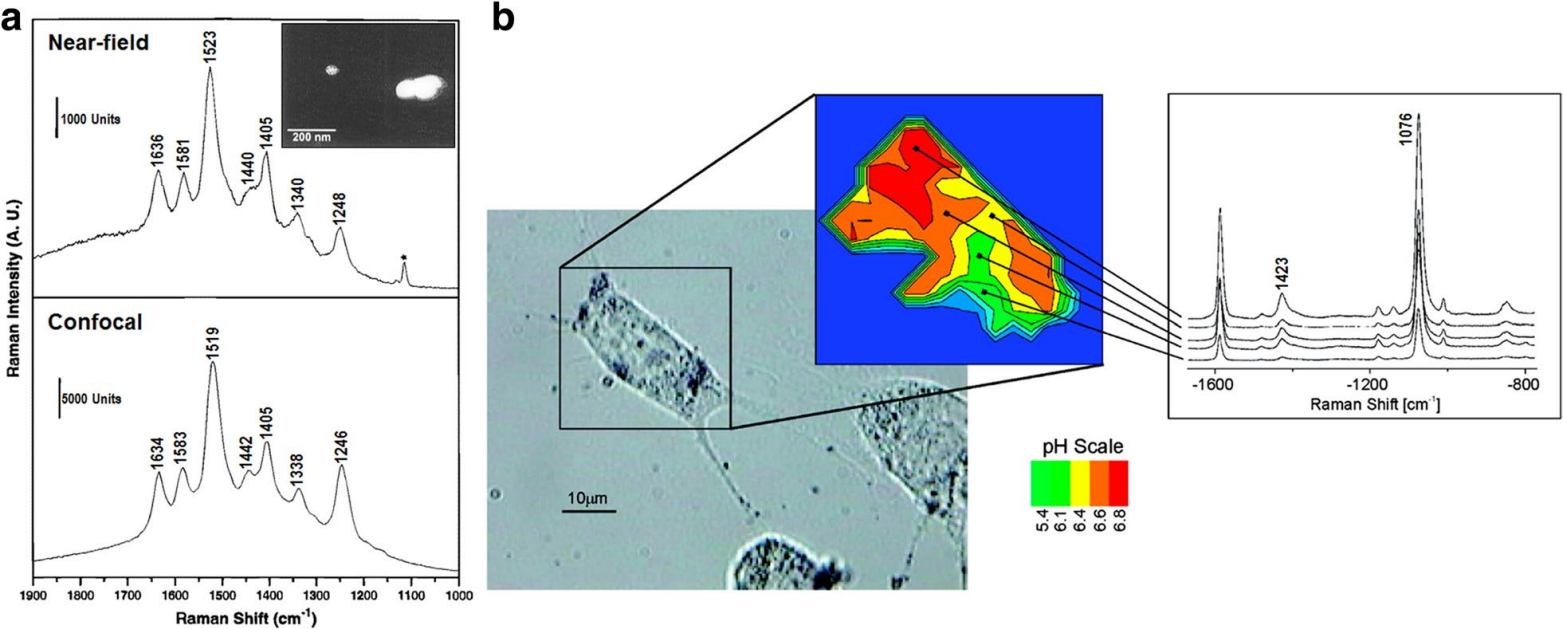
**a** Raman spectroscopic intensity of 3-hydroxykynurenine bound on silver nanoparticles captured by a near-field probe and a confocal microscope. An *inset figure* presents silver colloidal nanoparticles acquired by an atomic force microscope. The use of this figure from Ref. [64] is permitted by the American Chemical Society. **b** Intracellular pH value imaging of in vitro cultured cells using a nanoscale sensor consisting of 4-mercaptobenzoic acid functionalized gold nanoparticles. This figure from [67] is re-published with the permission of the American Chemical Society

Derived from Raman scattering, which is an inelastic photonic scattering coupled with the vibration mode in materials [58], Raman spectroscopy was designed to analyse the molecules that make up a specimen [59, 60]. In the first stage of Raman spectroscopy based molecular analysis, the application was limited because the probability of Raman scattering is significantly (1 in 10^7^) lower than that of the scattering of photons with the photon energy converging. Recently, several research groups discovered a highly enhanced intensity (10^3^–10^8^) of Raman scattering in the vicinity of metallic nanoparticles or nanosubstrates, which is termed surface-enhanced Raman scattering (SERS). SERS-based molecular analysis has been actively employed in various biological and chemical studies [61–63]. Emory and Nie investigated a near-field microscopic system to employ SERS on a single silver nanoparticle, and a highly improved sensitivity of molecular detection was confirmed by the measurements of Rhodamine 6G and 3-hydroxykynurenine attached onto the silver nanoparticles [64] in Fig. 2a. On the other hand, several research groups developed Raman spectroscopic pH measurement methods using nanoparticles functionalized by 4-mercaptobenzoic acid (pMBA), which have two chemical structure modes (OH and O^−^) changed by environmental pH values sensitively [65]. Talley et al. developed pMBA-functionalized silver nanoparticles to implement SERS-based intracellular pH sensing probes [66]. In the Raman spectroscopic test, these pH probes were responsive to pH changes from 6 to 8, and when inserted into Chinese hamster ovary (CHO) cells, they provided accurate intracellular pH information. Kneipp et al. designed and produced surface-enhanced hyper-Raman spectroscopy based intracellular pH nanoscale sensors for the optical pH imaging of cells [67]. The pH nanoscale sensor consisted of pMBA and gold nanoparticles, and the pH value image of an individual CHO cell was acquired using the pH nanosensor as shown in Fig. 2b. Wang et al. investigated a substrate consisting of silver nanoparticles and nanoscale anodic aluminium oxide channels for the enhancement of molecular detection by Raman spectroscopy [68].

### Nanoparticle-based fluorescence enhancements and their applications

**Fig. 3:**
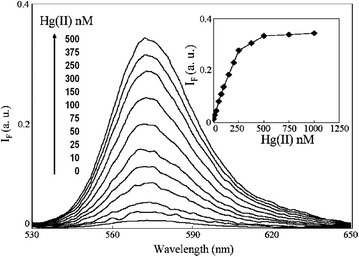
Fluorescence spectra of gold nanoparticles conjugated with Rhodamine B molecules and mercaptopropionic acid (MPA) upon exposure to Hg(II) at different concentrations. An *inset graph* indicates the fluorescence intensity changes in a peak wavelength (λ = 575 nm) under various Hg(II) concentrations. This figure from [72] is re-printed with the permission of the American Chemical Society

According to the explanation of LSPR in metallic nanoparticles in the session of “LSPR biosensors based on nanoparticles”, nanoparticles act as optical antennas that concentrate and localize the electromagnetic fields of the incident light with appropriate wavelengths. These concentrated and enhanced electromagnetic fields can excite fluorescent molecules with highly improved fluorescence efficiency. Although the improvement of the fluorescence efficiency is not proportional to the optical near-field enhancement due to non-radiative energy transfer, metallic nanoparticle induced plasmonic fluorescence enhancements have been applied to highly sensitive detection and fluorescence imaging. Anger and co-researchers presented calculations and experimental investigations of fluorescence enhancements obtained by the LSPR of a gold or silver nanoparticle with visible laser radiation [69, 70]. On a metallic nanoparticle using laser radiation with λ = 637 nm, a 10-fold improvement in the fluorescence yield was demonstrated. Kühn et al. also demonstrated gold nanoparticle based plasmonic fluorescence enhancements by varying the displacement between the molecule and the nanoparticle [71]. Huang and Chang developed high-performance fluorescence chemical sensors to measure Hg(II) using gold nanoparticles conjugated with Rhodamine B molecules and mercaptopropionic acid (MPA), as illustrated in Fig. 3 [72]. In the optimized environment, the sensitivity of the Hg(II) sensors using the fluorescence enhancement induced by the nanoparticles was 50-times improved. Li et al. suggested a high-sensitive Immunoglobulin E (IgE) detection method based on the improvement of fluorescence using nanoscale probes consisting of a silver nanoparticle, an aptamer, an oligomer and a fluorescent indicator (Cy3) [73]. These newly investigated nanoscale probes offer remarkable sensitivity and an improvement in the detection limit (40 pg/mL). Cheng et al. [74] applied gold nanoparticles and their near-field fluorescence enhancements to measure fluorescently-labelled deoxyribonucleic acid (DNA) probes with highly improved sensitivity. Taton et al. suggested an analytical method of combinational DNA arrays using modified gold nanoparticles and their fluorescence enhancements [75]. Ai et al. introduced ultrasensitive *Bacillus anthracis* indicators based on fluorescence improvements using europium nanoparticles [76]. These applications in biomedical engineering present the feasibility of early-stage diagnosis systems and nano-medicines.

**Fig. 4:**
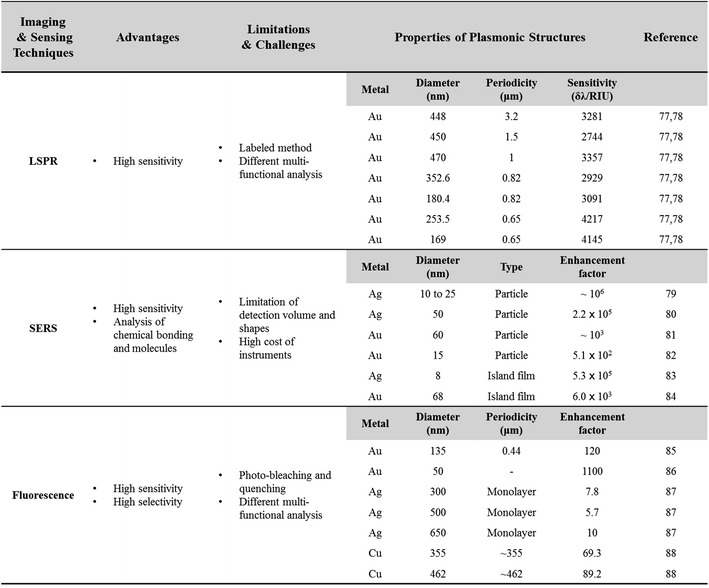
Advantages and limitations and challenges of three optical sensing and imaging techniques (LSPR, Raman spectroscopic measurement and fluorescence) using nanoparticles. Also, we represent a summary of the properties of plasmonic nanoparticles for sensing and imaging techniques

Before the description of recent developments in optical sensing and imaging techniques using advanced nanoparticles, Fig. 4 summarized the advantages of three optical sensing and imaging techniques (LSPR, Raman spectroscopic measurement and fluorescence) as well as the challenges, and also provides a summary of the properties of plasmonic nanoparticles for sensing and imaging techniques [77–88].

## Recent advances in optical sensing and imaging using novel nanoparticles

Although metallic nanoparticles suggest the improvement of the photonic performance by plasmonic field localizations and have been applied to various sensing and imaging applications, as introduced in the previous chapter, significantly higher enhancements by optimized field localizations for infinitesimal molecule detections and biocompatibility improvements for both in vitro and in vivo applications are needed. In this session, we briefly introduced several approaches for higher improvements in optical localized fields and biocompatibility.

### Specialized single-material nanoparticles for higher photonic enhancements

**Fig. 5:**
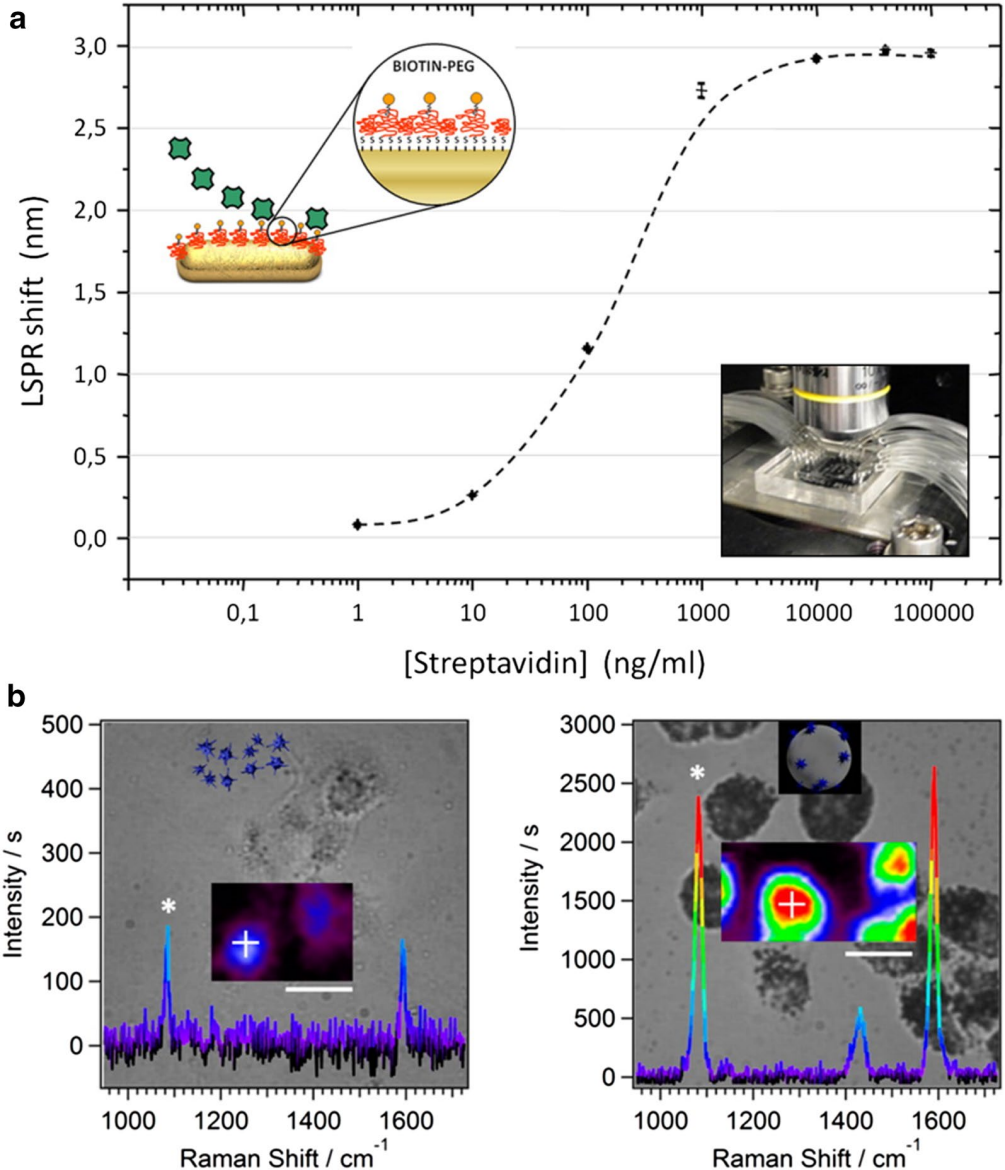
**a** Streptavidin measurements based on an LSPR microfluidic device using biotin-bound gold nanorods. An *inset figure* illustrates the microfluidic device on an optical spectroscopic measurement setup for metallic nanorod based high-performance molecule sensing. The re-print of this figure from [93] was permitted by the American Chemical Society. **b** High-sensitivity intracellular pH SERS imaging of A549 cells by polystyrene microbeads that were surrounded by 4-mercaptobenzoic acid conjugated gold nanostars. Compared to the use of 4-mercaptobenzoic acid conjugated gold nanostars without the polystyrene beads, the intracellular SERS signals that indicate the pH information in the compartments of the cells were enhanced. The use of this figure from [100] was permitted by the American Chemical Society

In both theoretical feasibility and experimental confirmation studies, several research groups introduced metallic nanoparticles with specialized shapes that have a higher efficiency to concentrate electromagnetic fields in optical regions. Grillet et al. [89] studied plasmon coupling and enhancement in silver nanoscale cube dimers and the difference in the enhancement efficiency and extinction spectra between various rounding shapes in the dimers. Metallic nanorods have also been used for sensing and imaging applications with high sensitivity. Marinakos et al. [90] described a label-free sensor using gold nanorod induced plasmonic localizations and enhancements. The authors claimed that the gold nanorod based sensor had a lower limit of detection and higher sensitivity compared to the gold nanoparticle based sensors. The feasibility of the gold nanorods as high-sensitivity probes in sensing specific biological molecules was also studied [91], and Rosman et al. [92] introduced the implementation of metallic nanorods for label-free, high-speed, and multiplexed molecular sensors using highly enhanced plasmon resonance performances. In a biomedical application, Aćimović et al. investigated a microfluidic system with the integration of an LSPR detection platform and gold nanorods that were functionalized as a cancer marker for the early-stage in vitro diagnosis of cancers, as illustrated in Fig. 5a [93]. In imaging and Raman spectroscopic integrations, Durr et al. introduced two-photon tumour cell imaging using gold nanorods that were functionalized as a specific molecular tracer [94], and Mirza et al. [95] described the possibility of gold nanorods serving as a highly efficient platform for SERS molecular detections. Several researchers investigated metallic nanoparticles with sharp tips called nanostars to improve the localized electromagnetic field efficiency and applied them in developing high-sensitivity probes. Hao and co-researchers presented extremely localized electromagnetic fields and plasmonic enhancements on the tips of a metallic nanostar using theoretical studies and suggested the feasibility of high-performance molecular sensing applications [96, 97]. Dondapati et al. [98] developed gold nanostars for application in label-free biosensing platforms. A preliminary study of optical streptavidin sensing using biotin-functionalized nanostars indicated that the gold nanostars could be integrated into highly efficient biological and chemical sensors. Shiohara et al. presented gold nanostars with satellite clusters that were attached at the ends of the tips and their SERS signal enhancements, which can be employed in high-performance SERS sensors [99]. Serrano-Montes and co-researchers introduced bio-imaging probes of gold nanostar surrounded polystyrene microbeads and applied them to the in vitro SERS imaging of A549 cells with high signal improvements compared to imaging with only gold nanostars, as illustrated in Fig. 5b [100].

### Specialized multi-materials nanoparticles for higher photonic enhancements

**Fig. 6:**
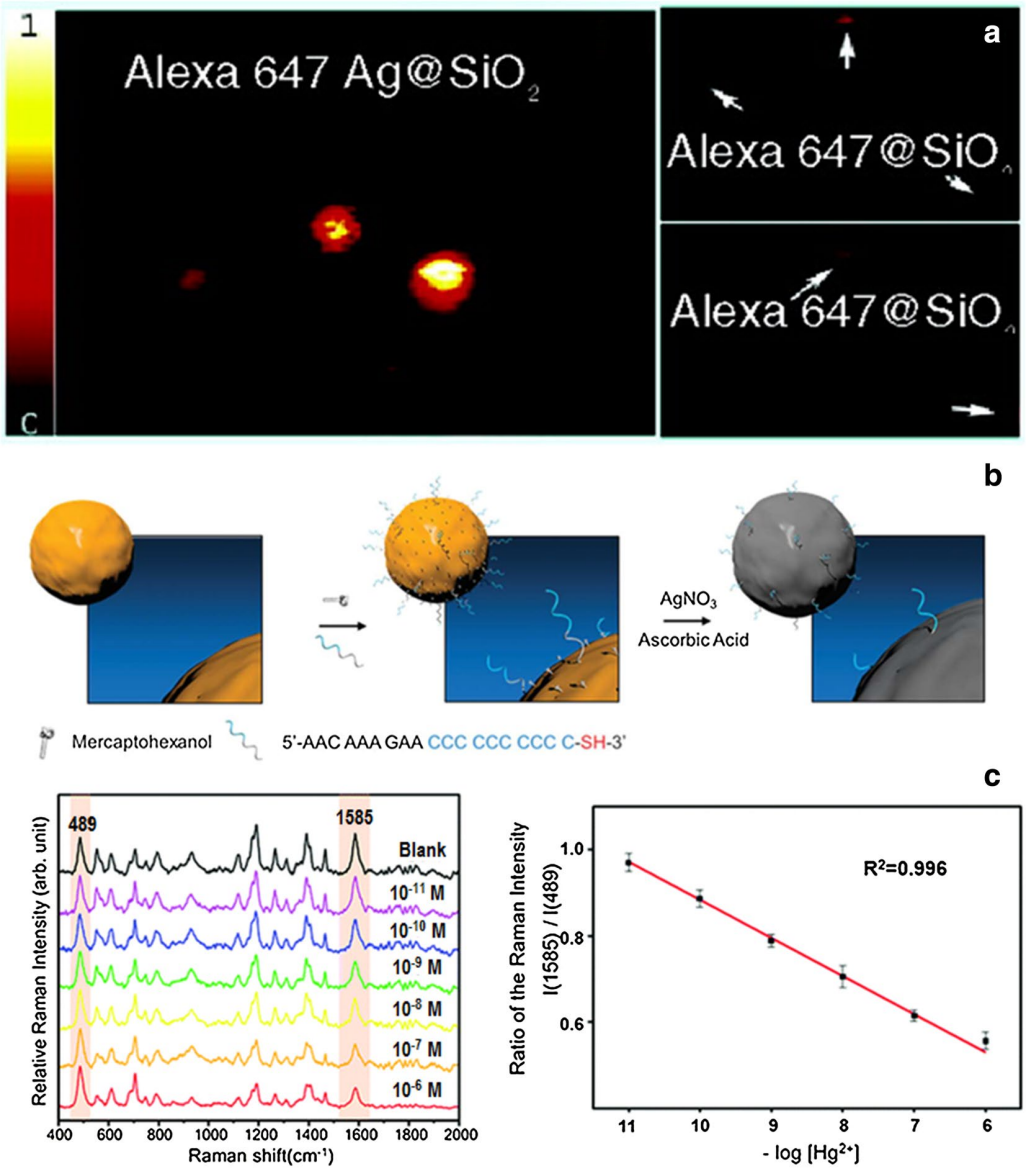
**a** Confocal fluorescence images of Alexa 647 tagged Ag-SiO_2_ core–shell and silica nanoparticles. A significant fluorescence enhancement of the Ag-SiO_2_ core–shell nanoparticles was observed. This figure from [103] is re-printed with the permission of the American Chemical Society. **b** Fabrication process of aptamer-attached Au–Ag core–shell nanoparticles for high-sensitivity measurements of Hg(II). **c** Surface-enhanced Raman spectroscopic intensities and peak intensity ratio between Cy3 and polydimethylsiloxane, the main material of the microfluidic device, under various Hg(II) concentrations. This figure from [107] is re-printed with the permission of the Royal Society of Chemistry

Multi-material nanoparticles have also been actively developed for enhanced sensitivity/fluorescence efficiency, multi-spectral responsibility, multi-functionalization and improved biocompatibility. For instance, Li et al. employed Au–Ag alloy nanoparticles to enhance the weak chemiluminescence from the oxidation processes between Rhodamine 6G and cerium(IV) [101]. Kim et al. investigated Au–Ag alloy nanoparticles with functionalization to react to *E. coli* and performed enhanced UV–VIS spectroscopic analysis using the nanoparticles [102]. Several research groups suggested and developed core–shell shaped multi-material nanoparticles in various applications with performance/functional improvements. Aslan and co-researchers demonstrated that Ag-SiO_2_ core–shell nanoparticles could be implemented in highly efficient fluorescence measuring and sensing platforms by a comparative fluorescence imaging study of Alexa 647 tagged Ag-SiO_2_ core–shell and silica nanoparticles, as illustrated in Fig. 6a [103]. Several synthesis and application studies described that iron oxide (Fe_2_O_3_)-Au core–shell nanoparticles can be employed in both high-sensitivity Raman spectroscopic measurements and effective separations using magnetic forces [104–106]. Chung and co-researchers investigated aptamer-attached Au–Ag core–shell nanoparticles for high-sensitivity measurements of Hg(II) based on plasmonic enhanced Raman spectroscopy in a microfluidic device, as illustrated in Fig. 6b [107].

### Surface engineering for biocompatibility and selectivity (should be reconsidered)

The surface property of nanoparticles plays an important role in a biocompatibility, bioavailability, selectivity, a stable dispersion and in vitro*/*vivo dynamics [108]. To successfully implement the nanoparticles for sensing and imaging applications, the nanoparticles require a biocompatible surface to exhibit their functionalities in the relevant biological solutions, and appropriate surface not only to bioconjugate with target biomolecules but also to impart the nanoparticles with “stealth” action to cross various undesired biological interactions [109]. Biocompatibility, has been defined as an acceptable functionality of the materials in a given biological environments, is basically linked to the hydrophilicity and the intrinsic toxicity of the as-prepared nanoparticle itself. The employment of hydrophilic materials to coat metallic nanoparticles increase stability and solubility with advantageous effects on toxicity and biocompatibility [110]. Several synthetic studies have been performed to replace the nature ligand on the surface of nanoparticles with small molecules (e.g. thiol, carboxyl, amine and phospine group), biomolecules and polymers. Gittins et al. reported the metallic nanoparticles could be functionalized by alkylthiol terminated molecules [111], and Isaacs et al. developed to change the surface property of gold nanoparticles from hydrophobic to hydrophilic using tetraoctylammonium bromide (TOAB) [112]. To overcome the poor stability of nanoparticle that covered by small molecules, Qian et al. used thiol-polyethylene glycol (thiol-PEG)-treated gold nanoparticles for the biocompatible and stable in vivo SERS detection and imaging of targeted tumour tissues [113]. Schofield et al. showed that glucose and sucrose have been used in reduction and stabilization agent of silver and gold nanoparticles [114]. Chung and co-researchers stated that Ag nanoparticles that were surface-modified using phosphoryl disulfides exhibited advanced biocompatibility [115].

All those above reports enable the synthesis and fabrication of nanoparticles with stability and biocompatibility. The recent advances to enhance the selectivity of sensing and imaging system in complex biological solutions can be categorized into two approaches: functionalization layers and biomolecules-mediated layers. The functionalization layers on surface of nanoparticles are commonly composed of numerous small molecules that interact with a specific target protein. For example, Pandya et al. utilized the boronic acid for selectively sensing the glucose [116]. The formation of functional carboxylic acids or amine group layers containing antibodies has usually been adapted for a highly specific binding with a target antigen. Also, for more selectively detection of target molecules, the surface of nanoparticles has been decorated by DNA sequences [117] and DNA aptamers [92]. Since the aptamers have much smaller size than antibodies, resulting in dramatic enhancement of sensing and imaging performance [118], the DNA aptamers-decorated nanoparticles can be useful for detection of small molecules [119], cancer marker [120], DNA [121] and proteins [122].

### Convergence of nanostructures and nanoparticles

As described in the session of “Localized surface plasmon resonance biosensors based on nanoparticles”, metallic nanostructures can also localize and enhance optical electromagnetic fields. Various studies on sensing and imaging applications based on nanostructure-based plasmonic field enhancements have been reported [123–127]. Plasmonic co-localization and the additional improvement of the optical electromagnetic fields between nano-components with an appropriate distance were investigated by both a theoretical approach and an experimental confirmation by a few research groups [128, 129]. Plasmonic co-localization has also been employed to establish high-sensitive optical sensing techniques, as described below.

**Fig. 7:**
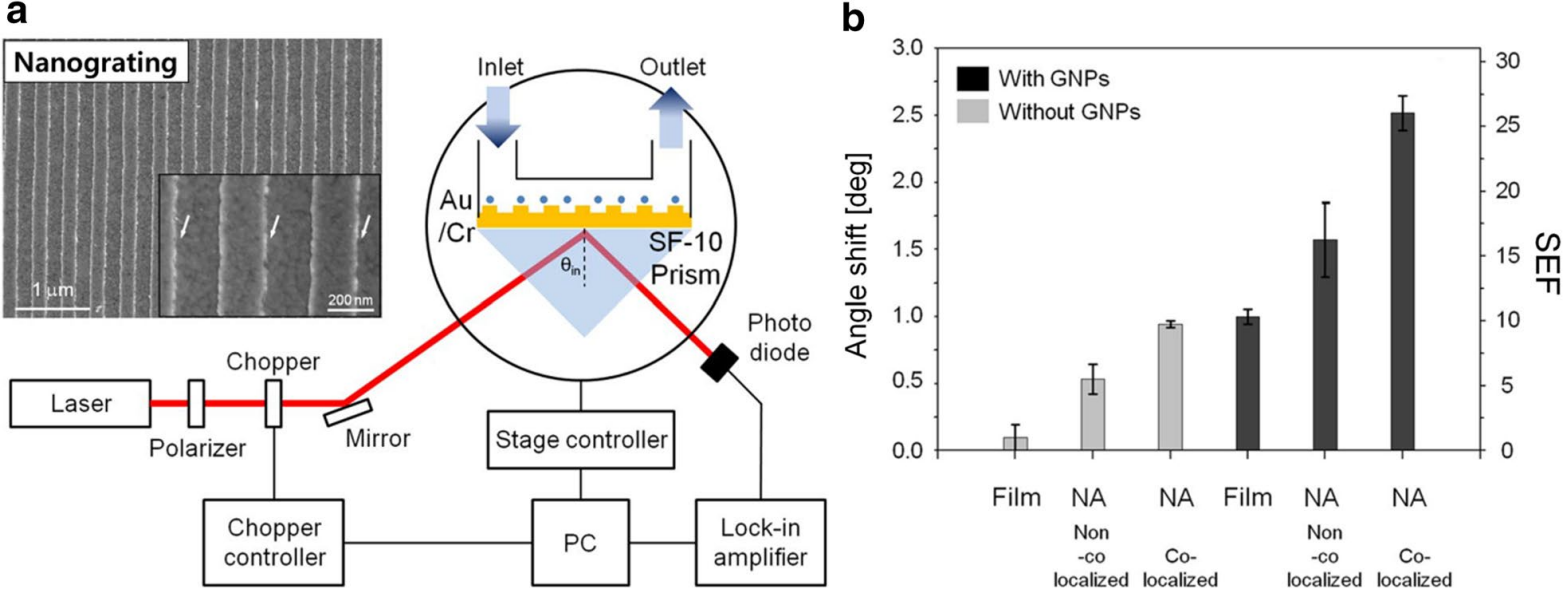
**a** Schematic of a surface plasmon resonance sensing configuration with a gold nanostructured grating and gold nanoparticles. **b** Resonance angle shift and sensitivity enhancement factor (SEF) measured under various conditions. The co-localization and it-based plasmonic enhancements provided 300-fold improvements of the sensitivity. This figure from [130] is re-printed with the permission of the Optical Society (OSA)

In the development of ultra-high sensitivity plasmonic sensors, Oh and co-researchers introduced an enhanced surface plasmon resonance sensor based on a co-localization between a nanostructured grating and a gold nanoparticle tagged to a biomolecule such as a DNA [130] (Fig. 7). Using a similar method, Kim et al. suggested a high-sensitivity biosensor using plasmonic co-localizations and it-based field enhancements between nanostructured gaps, which was fabricated by a specific area opening based on an angled metal-dielectric evaporation technique [131] and gold nanoparticle-tagged streptavidin molecules [132]. In the study of biotin–streptavidin binding and DNA detection [133], the biosensor presented the possibility of an investigation of hypersensitive chemical and biosensors. Ye and Van Dorpe introduced a phenomenon of field enhancements of plasmonic coupling on a gold nano-bowtie and a metallic nanoparticle between two gold nano-triangles in the nano-bowtie and demonstrated its feasibility in sensing applications [134].

**Fig. 8:**
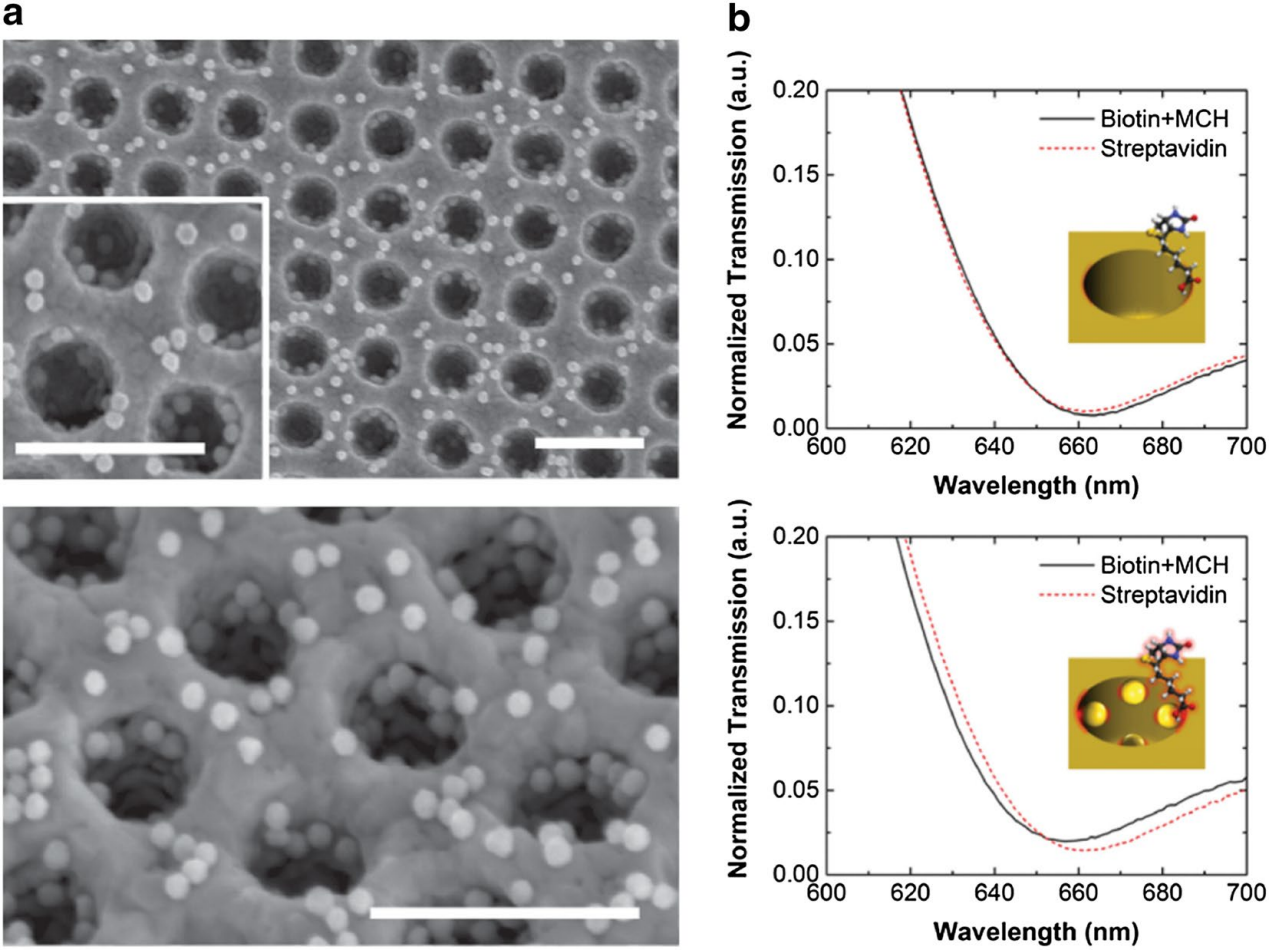
**a** Scanning electron microscope (SEM) images of a nanostructured Lycurgus cup array and gold nanoparticles. *Scale bar* in the figure indicates 0.5 μm. **b** Transmission spectral sensing of biotin–streptavidin binding on the nanostructured Lycurgus cup with/without gold nanoparticles. The resonance dip did not change in the 100 ng/mL biotin-streptavidin binding on the nano-Lycurgus cup without gold nanoparticles, but a spectral shift of 4.9 nm was detected on the nano-Lycurgus cup with gold nanoparticles. The re-print of this figure from [138] is permitted by Wiley

Also, several research groups studied the employment of the co-localization between the nanostructure and the nanoparticle to develop hypersensitive Raman spectroscopic measurements. Wei and co-researchers investigated the SERS spectra of nanoaperture-nanoparticle pairs using both theoretical and experimental approaches and suggested that the combination of nanoapertures and nanoparticles could offer ultrahigh-sensitivity SERS measurements of molecular activities such as DNA hybridizations [135]. Zhang et al. fabricated 3-dimensional nanostructured substrates that consisted of micron-sized silver pyramids and silver nanocubes for hypersensitive SERS detection [136]. Using parametric studies changing the shapes, sizes and inclination angles of the micron-sized structures on the substrate, the potential to establish hypersensitive SERS barcodes for detecting molecular activities was confirmed. Recently, Chang et al. developed a plasmon-enhanced SERS colorimetric sensor consisting of a nanostructured Lycurgus cup and nanoparticles in the cup, as illustrated in Fig. 8, and the feasibility of extraordinary transmission-based SERS ultrasensitive sensing applications was suggested [137, 138]. The possibility of enhanced Raman sensors using plasmonic electromagnetic enhancements based on the combination of a stochastic metallic nanostructure substrate and metallic nanoparticles was also recently reported [139].

## Concluding remarks

**Fig. 9:**
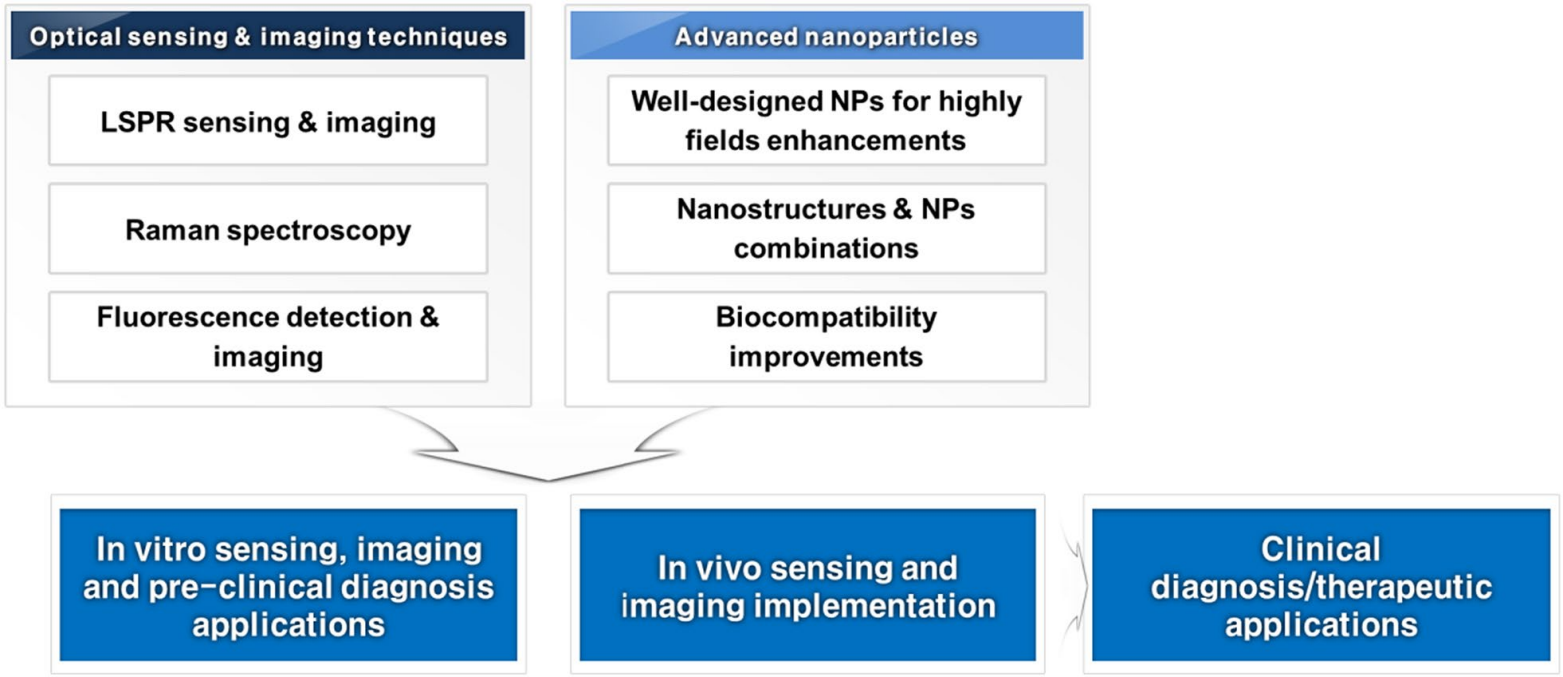
A diagram summary of the developments and applications of optical sensing/imaging techniques and nanoparticles

In this review article, we have described several current achievements of improved optical sensing and imaging applications using nanoparticles. Nanoparticles have been integrated to optical sensing and imaging techniques to achieve advanced detection performance for selected targets. Correlated to the development of optical sensing and imaging techniques, recent advances of nanoparticle-based optical sensing and imaging applications have progressed in two directions: (1) the investigation of well-designed and synthesized nanoparticles for extremely enhanced electromagnetic fields in optical regions and (2) the combination of nanostructures and nanoparticles to generate gap plasmonic localization for enhancing the fields, as illustrated in Fig. 9. Also, several research groups have developed nanoparticles with improved biocompatibility of that can be used for in vivo studies and clinical diagnosis/therapeutic implementations.

The integration and convergence of cutting-edge technologies in other fields were also expected. For example, optofluidic systems, which consist of microfluidic devices and optical sensing/imaging platforms [140, 141], can be applied in in vitro early-stage diagnosis with the integration of well-designed nanoparticles for the high-sensitivity detection of factors in specific diseases. High-resolution imaging techniques with breaking diffraction limits can support to develop various functionalized nanoparticles and can be applied to chemical, biological and biomedical analysis platforms [142–144]. Novel materials such as carbon nanotubes can be integrated in the development of photonic sensing and imaging techniques with highly improved performance [145]. These convergent studies raise the possibility of using nanoparticles practically and offer hope for discovers in these newly investigated research and application areas.
